# Shigella sonnei

**DOI:** 10.1016/j.tim.2020.02.011

**Published:** 2020-04-08

**Authors:** Vincenzo Torraca, Kathryn Holt, Serge Mostowy

**Affiliations:** 1 Department of Infection Biology, Faculty of Infectious and Tropical Diseases, London School of Hygiene and Tropical Medicine, London, UK; 2 Department of Infectious Diseases, Central Clinical School, Monash University, Melbourne, Australia

*Shigella sonnei* is a rod-shaped, Gram-negative facultative intracellular pathogen. It was named ‘Sonne’s bacillus’ after Carl Olaf Sonne who described it as a causative agent of bacillary dysentery. *S. sonnei* is distributed worldwide and represents the most common cause of shigellosis in industrialized regions in Europe, North America, and Australia. It is currently undergoing expansion in middle-income countries across Asia, Latin America, and the Middle East. *S. sonnei* evolved from *Escherichia coli* to specialize in intracellular infection of the human gut epithelium, and its genome comprises a 4.99 Mbp circular chromosome and a 216 kbp invasion plasmid (pINV) required for virulence. The chromosome is ~6% smaller than other *E. coli* and is punctuated by >300 copies of insertion sequence (IS) elements, whose expansion has degraded the genome through disruption and deletion of genes. Here we describe the key and disease facts allowing bacteria to evade host immune defences and to establish infection.

## Supplementary Material

Supplementalinformationassociatedwiththisarticlecanbefoundonlineat https://doi.org/10.1016/j.tim.2020.02.011.

Table S1

## Figures and Tables

**Figure 1:**
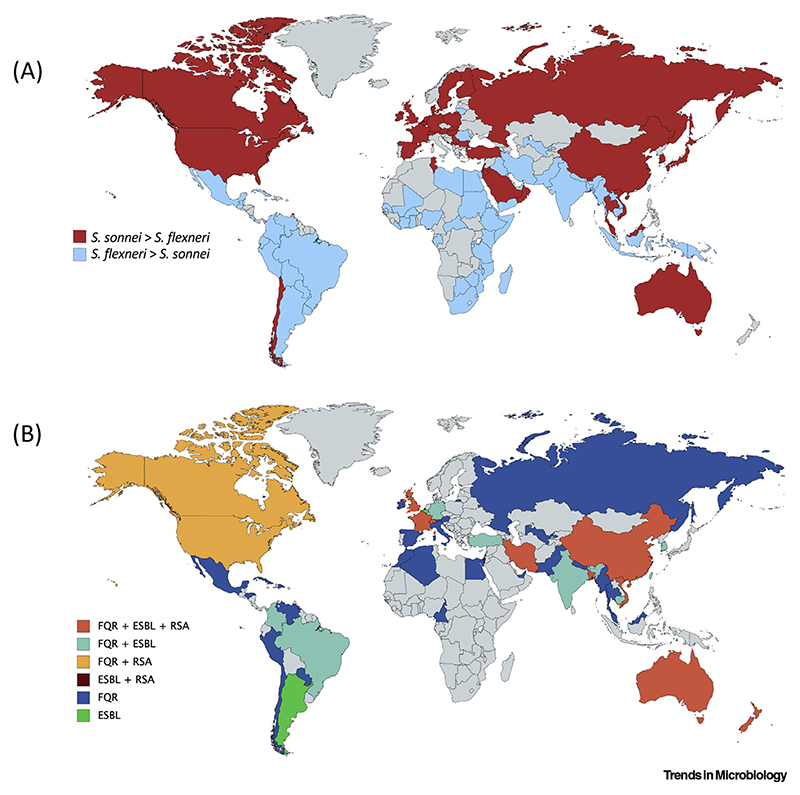
Epidemiology of *Shigella sonnei*. (A) Cases of *S. sonnei* versus *Shigella flexneri*. Cases of *S. sonnei* are increasing globally. Red indicates countries where *S. sonnei* is the dominant cause of shigellosis (when compared with *S. flexneri*). Blue indicates countries where a higher proportion of *S. flexneri* is still being reported (although *S. sonnei* cases may be rapidly increasing). Data presented according to Thompson *et al*. (see Literature). (B) Distribution of *S. sonnei* resistance to first- and second-line antibiotics. FQR, fluoroquinolone resistance; ESBL, extended-spectrum β-lactamase-producing (i.e., resistant to third-generation cephalosporins or carbapenems); RSA, reduced sensitivity to azithromycin. Countries are colored according to antibiotic-resistant cases reported in Table S1 in the supplemental information online.

**Figure 2:**
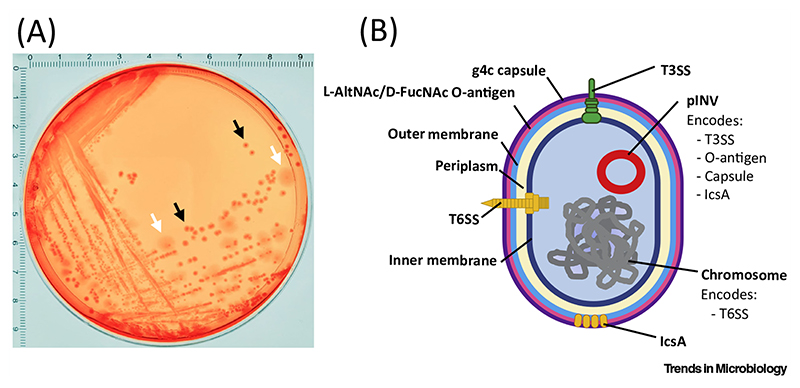
*Shigella sonnei* Virulence Determinants. (A) *S. sonnei* 53G platedon Congo Red agar.Thevirulenceplasmid (pINV) of *S. sonnei* is essential for pathogenesis *in vivo* but is unstable outside the host (and is lost when bacteria are cultured *in vitro*). As a result of maintenance or loss of pINV, *S. sonnei* presents two different phenotypes on Congo Red plates. Black arrows indicate small smooth red colonies (Phase I *S. sonnei*). These colonies retain pINV and accumulate Congo Red dye because they express a type III secretion system (T3SS). The small smooth phenotype is due to expression of O-antigen (O-Ag). White arrows indicate large rough white colonies (Phase II *S. sonnei*). These colonies are unable to accumulate Congo Red and have an irregular shape because they lose pINV (which encodes both T3SS and O-Ag). In this case, bacteria were plated from a glycerol stock (originally obtained from a liquid culture of a single Phase I colony) and incubated overnight at 37°C. Grid numbers indicate the size in centimetres. (B) Schematic representing key *S. sonnei* virulence determinants. *S. sonnei* encodes a T3SS crucial for host cell invasion; IcsAwhich mediates actin-based motility; aT6SS crucial for bacterial competition and niche occupancy; a horizontally acquired g4c capsule and O-antigen involved in resistance to phagocytosis, complement-mediated lysis, and phagolysosomal degradation. L-AltNAc, 2-acetamido-2-deoxy-L-altruronic acid; D-FucNAc, *N*-acetyl-2-acetamido-4-amino-2,4-dideoxy-D-fucose. Figure adapted from Torraca et al. (see Literature).

## Key Facts

A chromosomally encoded type VI secretion system (T6SS), involved in bacterial competition, is a key determinant for niche occupancy.

pINV-encoded virulence determinants include a type III secretion system (T3SS) involved in host cell invasion and phagosomal escape, the actin-polymerizing factor IcsA, a g4c capsule, and an unusual O-antigen encoded by genes horizontally acquired from *Plesiomonas shigelloides*.

The g4c capsule and O-antigen of *S. sonnei* protect the bacteria from a wide variety of host defence mechanisms, including phagocytosis, phagolysosome degradation, and complement-mediated lysis.

## Disease Facts

*S. sonnei* causes acute, self-limiting disease characterized by bloody diarrhea, fever, and abdominal pain. Infections can be life-threatening for children under 5 years and can stunt growth.

*S. sonnei* spreads via the fecal-oral route, as ingested bacteria can survive the gastric acidity and be released in stool.

Outbreaks are often centered around large events or care facilities. *S. sonnei* is also sexually transmitted, particularly among men who have sex with men.

The main treatment for bacillary dysentery is rehydration therapy. Fluoroquinolones, cephalosporins, and azithromycin are recommended when antimicrobials are required. However, resistance is increasingly common and new antimicrobials are needed.

There is no licensed vaccine. O-antigen-based immunization has been proposed; it holds particular promise as *S. sonnei* has only one serotype.

## Taxonomy and Classification

**KINGDOM:** Bacteria

**PHYLUM:** Proteobacteria

**CLASS:** Gammaproteobacteria

**ORDER:** Enterobacteriales

**FAMILY:** Enterobacteriaceae

**GENUS:** *Shigella* (whole-genome comparative analysis does not support distinct genus designation and suggests placement of *Shigella* spp. as lineages within the species *Escherichia coli*)

**SPECIES:** *Shigella sonnei*

**SUBSPECIES:** *S. sonnei* is monoclonal. All circulating strains originated from a common ancestor in Europe ~1500 AD. There is only one serogroup (*Shigella* serogroup D) consisting ofone serotype. Five distinct subtypes (lineages I-V) have been identified by whole-genome sequencing; the common laboratory strain 53G belongs to lineage II; however, most clinical isolates now belong to lineage III.
