# Supplementary material for: Shigella sonnei
Source: Trends Microbiol. Author manuscript; Available in PMC 2021 Jun 22. (PMC7611039; doi:10.1016/j.tim.2020.02.011)
Supplement: Table S1 [file EMS127852-supplement-Table_S1.docx]

**Table S1. Country-specific references for *S. sonnei* resistance to first- and second- line antibiotics.** (Listed in alphabetical order by country)

**Fluoroquinolone resistance**

| **Country** | **Reference** |
| --- | --- |
| **Algeria** | Chung The H, Boinett C, Pham Thanh D, Jenkins C, Weill FX, Howden BP, Valcanis M, De Lappe N, Cormican M, Wangchuk S, Bodhidatta L, Mason CJ, Nguyen TNT, Ha Thanh T, Voong VP, Duong VT, Nguyen PHL, Turner P, Wick R, Ceyssens PJ, Thwaites G, Holt KE, Thomson NR, Rabaa MA, Baker S. Dissecting the molecular evolution of fluoroquinolone-resistant *Shigella sonnei*. Nat Commun. 2019 Oct 23;10(1):4828. doi: 10.1038/s41467-019-12823-0. PubMed PMID: 31645551; PubMed Central PMCID: PMC6811581. |
| **Australia** | Chung The H, Boinett C, Pham Thanh D, Jenkins C, Weill FX, Howden BP, Valcanis M, De Lappe N, Cormican M, Wangchuk S, Bodhidatta L, Mason CJ, Nguyen TNT, Ha Thanh T, Voong VP, Duong VT, Nguyen PHL, Turner P, Wick R, Ceyssens PJ, Thwaites G, Holt KE, Thomson NR, Rabaa MA, Baker S. Dissecting the molecular evolution of fluoroquinolone-resistant *Shigella sonnei*. Nat Commun. 2019 Oct 23;10(1):4828. doi: 10.1038/s41467-019-12823-0. PubMed PMID: 31645551; PubMed Central PMCID: PMC6811581.  Chung The H, Rabaa MA, Pham Thanh D, De Lappe N, Cormican M, Valcanis M, Howden BP, Wangchuk S, Bodhidatta L, Mason CJ, Nguyen Thi Nguyen T, Vu Thuy D, Thompson CN, Phu Huong Lan N, Voong Vinh P, Ha Thanh T, Turner P, Sar P, Thwaites G, Thomson NR, Holt KE, Baker S. South Asia as a reservoir for the global spread of ciprofloxacin-resistant *Shigella sonnei*: A Cross-Sectional Study. PLoS Med. 2016 Aug 2;13(8):e1002055. doi: 10.1371/journal.pmed.1002055. PubMed PMID: 27483136; PubMed Central PMCID: PMC4970813.  Ingle DJ, Easton M, Valcanis M, Seemann T, Kwong JC, Stephens N, Carter GP, Gonçalves da Silva A, Adamopoulos J, Baines SL, Holt KE, Chow EPF, Fairley CK, Chen MY, Kirk MD, Howden BP, Williamson DA. Co-circulation of multidrug-resistant *Shigella* among men who have sex with men in Australia. Clin Infect Dis. 2019 Oct 15;69(9):1535-1544. doi: 10.1093/cid/ciz005. PubMed PMID: 30615105. |
| **Austria** | Chung The H, Baker S. Out of Asia: the independent rise and global spread of fluoroquinolone-resistant *Shigella*. Microb Genom. 2018 Apr;4(4). doi: 10.1099/mgen.0.000171. PubMed PMID: 29595412; PubMed Central PMCID: PMC5989582. |
| **Bangladesh** | Ud-Din AI, Wahid SU, Latif HA, Shahnaij M, Akter M, Azmi IJ, Hasan TN, Ahmed D, Hossain MA, Faruque AS, Faruque SM, Talukder KA. Changing trends in the prevalence of *Shigella* species: emergence of multi-drug resistant *Shigella sonnei* biotype g in Bangladesh. PLoS One. 2013 Dec 18;8(12):e82601. doi: 10.1371/journal.pone.0082601. eCollection 2013. PubMed PMID: 24367527; PubMed Central PMCID: PMC3867351.  Chung The H, Boinett C, Pham Thanh D, Jenkins C, Weill FX, Howden BP, Valcanis M, De Lappe N, Cormican M, Wangchuk S, Bodhidatta L, Mason CJ, Nguyen TNT, Ha Thanh T, Voong VP, Duong VT, Nguyen PHL, Turner P, Wick R, Ceyssens PJ, Thwaites G, Holt KE, Thomson NR, Rabaa MA, Baker S. Dissecting the molecular evolution of fluoroquinolone-resistant *Shigella sonnei*. Nat Commun. 2019 Oct 23;10(1):4828. doi: 10.1038/s41467-019-12823-0. PubMed PMID: 31645551; PubMed Central PMCID: PMC6811581. |
| **Bhutan** | Chung The H, Boinett C, Pham Thanh D, Jenkins C, Weill FX, Howden BP, Valcanis M, De Lappe N, Cormican M, Wangchuk S, Bodhidatta L, Mason CJ, Nguyen TNT, Ha Thanh T, Voong VP, Duong VT, Nguyen PHL, Turner P, Wick R, Ceyssens PJ, Thwaites G, Holt KE, Thomson NR, Rabaa MA, Baker S. Dissecting the molecular evolution of fluoroquinolone-resistant *Shigella sonnei*. Nat Commun. 2019 Oct 23;10(1):4828. doi: 10.1038/s41467-019-12823-0. PubMed PMID: 31645551; PubMed Central PMCID: PMC6811581.  Chung The H, Rabaa MA, Pham Thanh D, De Lappe N, Cormican M, Valcanis M, Howden BP, Wangchuk S, Bodhidatta L, Mason CJ, Nguyen Thi Nguyen T, Vu Thuy D, Thompson CN, Phu Huong Lan N, Voong Vinh P, Ha Thanh T, Turner P, Sar P, Thwaites G, Thomson NR, Holt KE, Baker S. South Asia as a reservoir for the global spread of ciprofloxacin-resistant *Shigella sonnei*: A Cross-Sectional Study. PLoS Med. 2016 Aug 2;13(8):e1002055. doi: 10.1371/journal.pmed.1002055. PubMed PMID: 27483136; PubMed Central PMCID: PMC4970813.  Chung The H, Rabaa MA, Thanh DP, Ruekit S, Wangchuk S, Dorji T, Tshering KP, Nguyen TNT, Vinh PV, Thanh TH, Minh CNN, Turner P, Sar P, Thwaites G, Holt KE, Thomson NR, Bodhidatta L, Jeffries Mason C, Baker S. Introduction and establishment of fluoroquinolone-resistant *Shigella sonnei* into Bhutan. Microb Genom. 2015 Dec 24;1(6):e000042. doi: 10.1099/mgen.0.000042. PubMed PMID: 28348825; PubMed Central PMCID: PMC5320628. |
| **Brazil** | Sati HF, Bruinsma N, Galas M, Hsieh J, Sanhueza A, Ramon Pardo P, Espinal MA. Characterizing *Shigella* species distribution and antimicrobial susceptibility to ciprofloxacin and nalidixic acid in Latin America between 2000-2015. PLoS One. 2019 Aug 2;14(8):e0220445. doi: 10.1371/journal.pone.0220445. PubMed PMID: 31374081; PubMed Central PMCID: PMC6677304.  Freitas DY, Araújo S, Folador ARC, Ramos RTJ, Azevedo JSN, Tacão M, Silva A, Henriques I, Baraúna RA. Extended spectrum beta-lactamase-producing Gram-negative bacteria recovered from an Amazonian lake near the city of Belém, Brazil. Front Microbiol. 2019 Feb 28;10:364. doi: 10.3389/fmicb.2019.00364. PubMed PMID: 30873145; PubMed Central PMCID: PMC6403167. |
| **Cambodia** | Chung The H, Boinett C, Pham Thanh D, Jenkins C, Weill FX, Howden BP, Valcanis M, De Lappe N, Cormican M, Wangchuk S, Bodhidatta L, Mason CJ, Nguyen TNT, Ha Thanh T, Voong VP, Duong VT, Nguyen PHL, Turner P, Wick R, Ceyssens PJ, Thwaites G, Holt KE, Thomson NR, Rabaa MA, Baker S. Dissecting the molecular evolution of fluoroquinolone-resistant *Shigella sonnei*. Nat Commun. 2019 Oct 23;10(1):4828. doi: 10.1038/s41467-019-12823-0. PubMed PMID: 31645551; PubMed Central PMCID: PMC6811581.  Chung The H, Rabaa MA, Pham Thanh D, De Lappe N, Cormican M, Valcanis M, Howden BP, Wangchuk S, Bodhidatta L, Mason CJ, Nguyen Thi Nguyen T, Vu Thuy D, Thompson CN, Phu Huong Lan N, Voong Vinh P, Ha Thanh T, Turner P, Sar P, Thwaites G, Thomson NR, Holt KE, Baker S. South Asia as a Reservoir for the Global Spread of Ciprofloxacin-Resistant *Shigella sonnei* A Cross-Sectional Study. PLoS Med. 2016 Aug 2;13(8):e1002055. doi: 10.1371/journal.pmed.1002055. PubMed PMID: 27483136; PubMed Central PMCID: PMC4970813.  Poramathikul K, Bodhidatta L, Chiek S, Oransathid W, Ruekit S, Nobthai P, Lurchachaiwong W, Serichantalergs O, Lon C, Swierczewski B. Multidrug-resistant *Shigella* infections in patients with diarrhea, Cambodia, 2014-2015. Emerg Infect Dis. 2016 Sep;22(9):1640-3. doi: 10.3201/eid2209.152058. PubMed PMID: 27532684; PubMed Central PMCID: PMC4994341. |
| **Cameroon** | Chung The H, Boinett C, Pham Thanh D, Jenkins C, Weill FX, Howden BP, Valcanis M, De Lappe N, Cormican M, Wangchuk S, Bodhidatta L, Mason CJ, Nguyen TNT, Ha Thanh T, Voong VP, Duong VT, Nguyen PHL, Turner P, Wick R, Ceyssens PJ, Thwaites G, Holt KE, Thomson NR, Rabaa MA, Baker S. Dissecting the molecular evolution of fluoroquinolone-resistant *Shigella sonnei*. Nat Commun. 2019 Oct 23;10(1):4828. doi: 10.1038/s41467-019-12823-0. PubMed PMID: 31645551; PubMed Central PMCID: PMC6811581. |
| **Canada** | Gaudreau C, Ratnayake R, Pilon PA, Gagnon S, Roger M, Lévesque S. Ciprofloxacin-resistant *Shigella sonnei* among men who have sex with men, Canada, 2010. Emerg Infect Dis. 2011 Sep;17(9):1747-50. doi: 10.3201/eid1709.102034. PubMed PMID: 21888811; PubMed Central PMCID: PMC3322076. |
| **Chile** | Sati HF, Bruinsma N, Galas M, Hsieh J, Sanhueza A, Ramon Pardo P, Espinal MA. Characterizing *Shigella* species distribution and antimicrobial susceptibility to ciprofloxacin and nalidixic acid in Latin America between 2000-2015. PLoS One. 2019 Aug 2;14(8):e0220445. doi: 10.1371/journal.pone.0220445. PubMed PMID: 31374081; PubMed Central PMCID: PMC6677304. |
| **China** | Zhang CL, Liu QZ, Wang J, Chu X, Shen LM, Guo YY. Epidemic and virulence characteristic of *Shigella* *spp*. with extended-spectrum cephalosporin resistance in Xiaoshan District, Hangzhou, China. BMC Infect Dis. 2014 May 15;14:260. doi: 10.1186/1471-2334-14-260. PubMed PMID: 24886028; PubMed Central PMCID: PMC4229937.  Chung The H, Boinett C, Pham Thanh D, Jenkins C, Weill FX, Howden BP, Valcanis M, De Lappe N, Cormican M, Wangchuk S, Bodhidatta L, Mason CJ, Nguyen TNT, Ha Thanh T, Voong VP, Duong VT, Nguyen PHL, Turner P, Wick R, Ceyssens PJ, Thwaites G, Holt KE, Thomson NR, Rabaa MA, Baker S. Dissecting the molecular evolution of fluoroquinolone-resistant *Shigella sonnei*. Nat Commun. 2019 Oct 23;10(1):4828. doi: 10.1038/s41467-019-12823-0. PubMed PMID: 31645551; PubMed Central PMCID: PMC6811581. |
| **Colombia** | Sati HF, Bruinsma N, Galas M, Hsieh J, Sanhueza A, Ramon Pardo P, Espinal MA. Characterizing *Shigella* species distribution and antimicrobial susceptibility to ciprofloxacin and nalidixic acid in Latin America between 2000-2015. PLoS One. 2019 Aug 2;14(8):e0220445. doi: 10.1371/journal.pone.0220445. PubMed PMID: 31374081; PubMed Central PMCID: PMC6677304. |
| **Cuba** | Sati HF, Bruinsma N, Galas M, Hsieh J, Sanhueza A, Ramon Pardo P, Espinal MA. Characterizing *Shigella* species distribution and antimicrobial susceptibility to ciprofloxacin and nalidixic acid in Latin America between 2000-2015. PLoS One. 2019 Aug 2;14(8):e0220445. doi: 10.1371/journal.pone.0220445. PubMed PMID: 31374081; PubMed Central PMCID: PMC6677304. |
| **Dominican Republic** | Sati HF, Bruinsma N, Galas M, Hsieh J, Sanhueza A, Ramon Pardo P, Espinal MA. Characterizing *Shigella* species distribution and antimicrobial susceptibility to ciprofloxacin and nalidixic acid in Latin America between 2000-2015. PLoS One. 2019 Aug 2;14(8):e0220445. doi: 10.1371/journal.pone.0220445. PubMed PMID: 31374081; PubMed Central PMCID: PMC6677304. |
| **Egypt** | Chung The H, Boinett C, Pham Thanh D, Jenkins C, Weill FX, Howden BP, Valcanis M, De Lappe N, Cormican M, Wangchuk S, Bodhidatta L, Mason CJ, Nguyen TNT, Ha Thanh T, Voong VP, Duong VT, Nguyen PHL, Turner P, Wick R, Ceyssens PJ, Thwaites G, Holt KE, Thomson NR, Rabaa MA, Baker S. Dissecting the molecular evolution of fluoroquinolone-resistant *Shigella sonnei*. Nat Commun. 2019 Oct 23;10(1):4828. doi: 10.1038/s41467-019-12823-0. PubMed PMID: 31645551; PubMed Central PMCID: PMC6811581. |
| **France** | Chung The H, Boinett C, Pham Thanh D, Jenkins C, Weill FX, Howden BP, Valcanis M, De Lappe N, Cormican M, Wangchuk S, Bodhidatta L, Mason CJ, Nguyen TNT, Ha Thanh T, Voong VP, Duong VT, Nguyen PHL, Turner P, Wick R, Ceyssens PJ, Thwaites G, Holt KE, Thomson NR, Rabaa MA, Baker S. Dissecting the molecular evolution of fluoroquinolone-resistant *Shigella sonnei*. Nat Commun. 2019 Oct 23;10(1):4828. doi: 10.1038/s41467-019-12823-0. PubMed PMID: 31645551; PubMed Central PMCID: PMC6811581. |
| **Germany** | Hoffmann C, Sahly H, Jessen A, Ingiliz P, Stellbrink HJ, Neifer S, Schewe K, Dupke S, Baumgarten A, Kuschel A, Krznaric I. High rates of quinolone-resistant strains of *Shigella sonnei* in HIV-infected MSM. Infection. 2013 Oct;41(5):999-1003. doi: 10.1007/s15010-013-0501-4. PubMed PMID: 23852945.  Chung The H, Boinett C, Pham Thanh D, Jenkins C, Weill FX, Howden BP, Valcanis M, De Lappe N, Cormican M, Wangchuk S, Bodhidatta L, Mason CJ, Nguyen TNT, Ha Thanh T, Voong VP, Duong VT, Nguyen PHL, Turner P, Wick R, Ceyssens PJ, Thwaites G, Holt KE, Thomson NR, Rabaa MA, Baker S. Dissecting the molecular evolution of fluoroquinolone-resistant *Shigella sonnei*. Nat Commun. 2019 Oct 23;10(1):4828. doi: 10.1038/s41467-019-12823-0. PubMed PMID: 31645551; PubMed Central PMCID: PMC6811581. |
| **Honduras** | Sati HF, Bruinsma N, Galas M, Hsieh J, Sanhueza A, Ramon Pardo P, Espinal MA. Characterizing *Shigella* species distribution and antimicrobial susceptibility to ciprofloxacin and nalidixic acid in Latin America between 2000-2015. PLoS One. 2019 Aug 2;14(8):e0220445. doi: 10.1371/journal.pone.0220445. PubMed PMID: 31374081; PubMed Central PMCID: PMC6677304. |
| **India** | Ghosh S, Pazhani GP, Chowdhury G, Guin S, Dutta S, Rajendran K, Bhattacharya MK, Takeda Y, Niyogi SK, Nair GB, Ramamurthy T. Genetic characteristics and changing antimicrobial resistance among Shigella spp. isolated from hospitalized diarrhoeal patients in Kolkata, India. J Med Microbiol. 2011 Oct;60(Pt10):1460-6. doi: 10.1099/jmm.0.032920-0. Epub 2011 Jun 9. PubMed PMID: 21659504.  Madhavan A, Balakrishnan S, Vasudevapanicker J. Antibiotic susceptibility pattern of *Shigella* isolates in a tertiary healthcare center. J Lab Physicians. 2018 Apr-Jun;10(2):140-144. doi: 10.4103/JLP.JLP_93_17. PubMed PMID: 29692577; PubMed Central PMCID: PMC5896178.  Chung The H, Boinett C, Pham Thanh D, Jenkins C, Weill FX, Howden BP, Valcanis M, De Lappe N, Cormican M, Wangchuk S, Bodhidatta L, Mason CJ, Nguyen TNT, Ha Thanh T, Voong VP, Duong VT, Nguyen PHL, Turner P, Wick R, Ceyssens PJ, Thwaites G, Holt KE, Thomson NR, Rabaa MA, Baker S. Dissecting the molecular evolution of fluoroquinolone-resistant *Shigella sonnei*. Nat Commun. 2019 Oct 23;10(1):4828. doi: 10.1038/s41467-019-12823-0. PubMed PMID: 31645551; PubMed Central PMCID: PMC6811581. |
| **Iran** | Khademi F, Sahebkar A. Fluoroquinolones-resistant *Shigella* species in Iranian children: a meta-analysis. World J Pediatr. 2019 Oct;15(5):441-453. doi: 10.1007/s12519-019-00263-1. Epub 2019 Jun 1. PubMed PMID: 31154582. |
| **Ireland** | Chung The H, Boinett C, Pham Thanh D, Jenkins C, Weill FX, Howden BP, Valcanis M, De Lappe N, Cormican M, Wangchuk S, Bodhidatta L, Mason CJ, Nguyen TNT, Ha Thanh T, Voong VP, Duong VT, Nguyen PHL, Turner P, Wick R, Ceyssens PJ, Thwaites G, Holt KE, Thomson NR, Rabaa MA, Baker S. Dissecting the molecular evolution of fluoroquinolone-resistant *Shigella sonnei*. Nat Commun. 2019 Oct 23;10(1):4828. doi: 10.1038/s41467-019-12823-0. PubMed PMID: 31645551; PubMed Central PMCID: PMC6811581.  Chung The H, Rabaa MA, Pham Thanh D, De Lappe N, Cormican M, Valcanis M, Howden BP, Wangchuk S, Bodhidatta L, Mason CJ, Nguyen Thi Nguyen T, Vu Thuy D, Thompson CN, Phu Huong Lan N, Voong Vinh P, Ha Thanh T, Turner P, Sar P, Thwaites G, Thomson NR, Holt KE, Baker S. South Asia as a Reservoir for the Global Spread of Ciprofloxacin-Resistant *Shigella sonnei* A Cross-Sectional Study. PLoS Med. 2016 Aug 2;13(8):e1002055. doi: 10.1371/journal.pmed.1002055. PubMed PMID: 27483136; PubMed Central PMCID: PMC4970813. |
| **Italy** | Chung The H, Boinett C, Pham Thanh D, Jenkins C, Weill FX, Howden BP, Valcanis M, De Lappe N, Cormican M, Wangchuk S, Bodhidatta L, Mason CJ, Nguyen TNT, Ha Thanh T, Voong VP, Duong VT, Nguyen PHL, Turner P, Wick R, Ceyssens PJ, Thwaites G, Holt KE, Thomson NR, Rabaa MA, Baker S. Dissecting the molecular evolution of fluoroquinolone-resistant *Shigella sonnei*. Nat Commun. 2019 Oct 23;10(1):4828. doi: 10.1038/s41467-019-12823-0. PubMed PMID: 31645551; PubMed Central PMCID: PMC6811581. |
| **Lebanon** | Chung The H, Boinett C, Pham Thanh D, Jenkins C, Weill FX, Howden BP, Valcanis M, De Lappe N, Cormican M, Wangchuk S, Bodhidatta L, Mason CJ, Nguyen TNT, Ha Thanh T, Voong VP, Duong VT, Nguyen PHL, Turner P, Wick R, Ceyssens PJ, Thwaites G, Holt KE, Thomson NR, Rabaa MA, Baker S. Dissecting the molecular evolution of fluoroquinolone-resistant *Shigella sonnei*. Nat Commun. 2019 Oct 23;10(1):4828. doi: 10.1038/s41467-019-12823-0. PubMed PMID: 31645551; PubMed Central PMCID: PMC6811581. |
| **Malaysia** | Chung The H, Baker S. Out of Asia: the independent rise and global spread of fluoroquinolone-resistant *Shigella*. Microb Genom. 2018 Apr;4(4). doi: 10.1099/mgen.0.000171. PubMed PMID: 29595412; PubMed Central PMCID: PMC5989582. |
| **Mexico** | Sati HF, Bruinsma N, Galas M, Hsieh J, Sanhueza A, Ramon Pardo P, Espinal MA. Characterizing *Shigella species* distribution and antimicrobial susceptibility to ciprofloxacin and nalidixic acid in Latin America between 2000-2015. PLoS One. 2019 Aug 2;14(8):e0220445. doi: 10.1371/journal.pone.0220445. PubMed PMID: 31374081; PubMed Central PMCID: PMC6677304. |
| **Morocco** | Chung The H, Boinett C, Pham Thanh D, Jenkins C, Weill FX, Howden BP, Valcanis M, De Lappe N, Cormican M, Wangchuk S, Bodhidatta L, Mason CJ, Nguyen TNT, Ha Thanh T, Voong VP, Duong VT, Nguyen PHL, Turner P, Wick R, Ceyssens PJ, Thwaites G, Holt KE, Thomson NR, Rabaa MA, Baker S. Dissecting the molecular evolution of fluoroquinolone-resistant *Shigella sonnei*. Nat Commun. 2019 Oct 23;10(1):4828. doi: 10.1038/s41467-019-12823-0. PubMed PMID: 31645551; PubMed Central PMCID: PMC6811581. |
| **Myanmar** | Chung The H, Boinett C, Pham Thanh D, Jenkins C, Weill FX, Howden BP, Valcanis M, De Lappe N, Cormican M, Wangchuk S, Bodhidatta L, Mason CJ, Nguyen TNT, Ha Thanh T, Voong VP, Duong VT, Nguyen PHL, Turner P, Wick R, Ceyssens PJ, Thwaites G, Holt KE, Thomson NR, Rabaa MA, Baker S. Dissecting the molecular evolution of fluoroquinolone-resistant *Shigella sonnei*. Nat Commun. 2019 Oct 23;10(1):4828. doi: 10.1038/s41467-019-12823-0. PubMed PMID: 31645551; PubMed Central PMCID: PMC6811581. |
| **Nepal** | Chung The H, Boinett C, Pham Thanh D, Jenkins C, Weill FX, Howden BP, Valcanis M, De Lappe N, Cormican M, Wangchuk S, Bodhidatta L, Mason CJ, Nguyen TNT, Ha Thanh T, Voong VP, Duong VT, Nguyen PHL, Turner P, Wick R, Ceyssens PJ, Thwaites G, Holt KE, Thomson NR, Rabaa MA, Baker S. Dissecting the molecular evolution of fluoroquinolone-resistant *Shigella sonnei*. Nat Commun. 2019 Oct 23;10(1):4828. doi: 10.1038/s41467-019-12823-0. PubMed PMID: 31645551; PubMed Central PMCID: PMC6811581. |
| **New Zealand** | Heffernan H, Woodhouse R, Hewison C, Sherwood J. Antimicrobial resistance among *Shigella* in New Zealand. N Z Med J. 2018 Jun 22;131(1477):56-62. PubMed PMID: 29927916. |
| **Pakistan** | Chung The H, Boinett C, Pham Thanh D, Jenkins C, Weill FX, Howden BP, Valcanis M, De Lappe N, Cormican M, Wangchuk S, Bodhidatta L, Mason CJ, Nguyen TNT, Ha Thanh T, Voong VP, Duong VT, Nguyen PHL, Turner P, Wick R, Ceyssens PJ, Thwaites G, Holt KE, Thomson NR, Rabaa MA, Baker S. Dissecting the molecular evolution of fluoroquinolone-resistant *Shigella sonnei*. Nat Commun. 2019 Oct 23;10(1):4828. doi: 10.1038/s41467-019-12823-0. PubMed PMID: 31645551; PubMed Central PMCID: PMC6811581. |
| **Paraguay** | Sati HF, Bruinsma N, Galas M, Hsieh J, Sanhueza A, Ramon Pardo P, Espinal MA. Characterizing *Shigella* species distribution and antimicrobial susceptibility to ciprofloxacin and nalidixic acid in Latin America between 2000-2015. PLoS One. 2019 Aug 2;14(8):e0220445. doi: 10.1371/journal.pone.0220445. PubMed PMID: 31374081; PubMed Central PMCID: PMC6677304. |
| **Peru** | Sati HF, Bruinsma N, Galas M, Hsieh J, Sanhueza A, Ramon Pardo P, Espinal MA. Characterizing *Shigella* species distribution and antimicrobial susceptibility to ciprofloxacin and nalidixic acid in Latin America between 2000-2015. PLoS One. 2019 Aug 2;14(8):e0220445. doi: 10.1371/journal.pone.0220445. PubMed PMID: 31374081; PubMed Central PMCID: PMC6677304.  Baker KS, Campos J, Pichel M, Della Gaspera A, Duarte-Martínez F, Campos-Chacón E, Bolaños-Acuña HM, Guzmán-Verri C, Mather AE, Diaz Velasco S, Zamudio Rojas ML, Forbester JL, Connor TR, Keddy KH, Smith AM, López de Delgado EA, Angiolillo G, Cuaical N, Fernández J, Aguayo C, Morales Aguilar M, Valenzuela C, Morales Medrano AJ, Sirok A, Weiler Gustafson N, Diaz Guevara PL, Montaño LA, Perez E, Thomson NR. Whole genome sequencing of *Shigella sonnei* through PulseNet Latin America and Caribbean: advancing global surveillance of foodborne illnesses. Clin Microbiol Infect. 2017 Nov;23(11):845-853. doi: 10.1016/j.cmi.2017.03.021. PubMed PMID: 28389276; PubMed Central PMCID: PMC5667938. |
| **Russia** | The interregional association for clinical microbiology and antimicrobial chemoterapy; <http://www.antibiotic.ru/iacmac/en/rosnet/report1.shtml> |
| **South Korea** | Kim JS, Kim JJ, Kim SJ, Jeon SE, Seo KY, Choi JK, Kim NO, Hong S, Chung GT, Yoo CK, Kim YT, Cheun HI, Bae GR, Yeo YH, Ha GJ, Choi MS, Kang SJ, Kim J. Outbreak of ciprofloxacin-resistant *Shigella sonnei* associated with travel to Vietnam, Republic of Korea. Emerg Infect Dis. 2015 Jul;21(7):1247-50. doi: 10.3201/eid2107.150363. PubMed PMID: 26079171; PubMed Central PMCID: PMC4480405. |
| **Spain** | Chung The H, Boinett C, Pham Thanh D, Jenkins C, Weill FX, Howden BP, Valcanis M, De Lappe N, Cormican M, Wangchuk S, Bodhidatta L, Mason CJ, Nguyen TNT, Ha Thanh T, Voong VP, Duong VT, Nguyen PHL, Turner P, Wick R, Ceyssens PJ, Thwaites G, Holt KE, Thomson NR, Rabaa MA, Baker S. Dissecting the molecular evolution of fluoroquinolone-resistant *Shigella sonnei*. Nat Commun. 2019 Oct 23;10(1):4828. doi: 10.1038/s41467-019-12823-0. PubMed PMID: 31645551; PubMed Central PMCID: PMC6811581. |
| **Switzerland** | Nüesch-Inderbinen M, Heini N, Zurfluh K, Althaus D, Hächler H, Stephan R. *Shigella* antimicrobial drug resistance mechanisms, 2004-2014. Emerg Infect Dis. 2016 Jun;22(6):1083-5. doi: 10.3201/eid2206.152088. PubMed PMID: 27191035; PubMed Central PMCID: PMC4880098. |
| **Taiwan** | Chiou CS, Izumiya H, Kawamura M, Liao YS, Su YS, Wu HH, Chen WC, Lo YC. The worldwide spread of ciprofloxacin-resistant *Shigella sonnei* among HIV-infected men who have sex with men, Taiwan. Clin Microbiol Infect. 2016 Apr;22(4):383.e11-383.e16. doi: 10.1016/j.cmi.2015.12.021. Epub 2016 Jan 20. PubMed PMID: 26806133. |
| **Thailand** | Chung The H, Boinett C, Pham Thanh D, Jenkins C, Weill FX, Howden BP, Valcanis M, De Lappe N, Cormican M, Wangchuk S, Bodhidatta L, Mason CJ, Nguyen TNT, Ha Thanh T, Voong VP, Duong VT, Nguyen PHL, Turner P, Wick R, Ceyssens PJ, Thwaites G, Holt KE, Thomson NR, Rabaa MA, Baker S. Dissecting the molecular evolution of fluoroquinolone-resistant *Shigella sonnei*. Nat Commun. 2019 Oct 23;10(1):4828. doi: 10.1038/s41467-019-12823-0. PubMed PMID: 31645551; PubMed Central PMCID: PMC6811581.  Chung The H, Rabaa MA, Pham Thanh D, De Lappe N, Cormican M, Valcanis M, Howden BP, Wangchuk S, Bodhidatta L, Mason CJ, Nguyen Thi Nguyen T, Vu Thuy D, Thompson CN, Phu Huong Lan N, Voong Vinh P, Ha Thanh T, Turner P, Sar P, Thwaites G, Thomson NR, Holt KE, Baker S. South Asia as a reservoir for the global spread of ciprofloxacin-resistant *Shigella sonnei*: A Cross-Sectional Study. PLoS Med. 2016 Aug 2;13(8):e1002055. doi: 10.1371/journal.pmed.1002055. PubMed PMID: 27483136; PubMed Central PMCID: PMC4970813. |
| **Turkey** | Chung The H, Boinett C, Pham Thanh D, Jenkins C, Weill FX, Howden BP, Valcanis M, De Lappe N, Cormican M, Wangchuk S, Bodhidatta L, Mason CJ, Nguyen TNT, Ha Thanh T, Voong VP, Duong VT, Nguyen PHL, Turner P, Wick R, Ceyssens PJ, Thwaites G, Holt KE, Thomson NR, Rabaa MA, Baker S. Dissecting the molecular evolution of fluoroquinolone-resistant *Shigella sonnei*. Nat Commun. 2019 Oct 23;10(1):4828. doi: 10.1038/s41467-019-12823-0. PubMed PMID: 31645551; PubMed Central PMCID: PMC6811581. |
| **United Arab Emirates** | Chung The H, Boinett C, Pham Thanh D, Jenkins C, Weill FX, Howden BP, Valcanis M, De Lappe N, Cormican M, Wangchuk S, Bodhidatta L, Mason CJ, Nguyen TNT, Ha Thanh T, Voong VP, Duong VT, Nguyen PHL, Turner P, Wick R, Ceyssens PJ, Thwaites G, Holt KE, Thomson NR, Rabaa MA, Baker S. Dissecting the molecular evolution of fluoroquinolone-resistant *Shigella sonnei*. Nat Commun. 2019 Oct 23;10(1):4828. doi: 10.1038/s41467-019-12823-0. PubMed PMID: 31645551; PubMed Central PMCID: PMC6811581. |
| **United Kingdom** | Chung The H, Boinett C, Pham Thanh D, Jenkins C, Weill FX, Howden BP, Valcanis M, De Lappe N, Cormican M, Wangchuk S, Bodhidatta L, Mason CJ, Nguyen TNT, Ha Thanh T, Voong VP, Duong VT, Nguyen PHL, Turner P, Wick R, Ceyssens PJ, Thwaites G, Holt KE, Thomson NR, Rabaa MA, Baker S. Dissecting the molecular evolution of fluoroquinolone-resistant *Shigella sonnei*. Nat Commun. 2019 Oct 23;10(1):4828. doi: 10.1038/s41467-019-12823-0. PubMed PMID: 31645551; PubMed Central PMCID: PMC6811581. |
| **United States** | Chung The H, Rabaa MA, Pham Thanh D, De Lappe N, Cormican M, Valcanis M, Howden BP, Wangchuk S, Bodhidatta L, Mason CJ, Nguyen Thi Nguyen T, Vu Thuy D, Thompson CN, Phu Huong Lan N, Voong Vinh P, Ha Thanh T, Turner P, Sar P, Thwaites G, Thomson NR, Holt KE, Baker S. South Asia as a reservoir for the global spread of ciprofloxacin-resistant *Shigella sonnei*: A Cross-Sectional Study. PLoS Med. 2016 Aug 2;13(8):e1002055. doi: 10.1371/journal.pmed.1002055. PubMed PMID: 27483136; PubMed Central PMCID: PMC4970813.  Kozyreva VK, Jospin G, Greninger AL, Watt JP, Eisen JA, Chaturvedi V. Recent outbreaks of shigellosis in California caused by two distinct populations of *Shigella sonnei* with either increased virulence or fluoroquinolone resistance. mSphere. 2016 Nov-Dec; 1(6): e00344-16. P doi: 10.1128/mSphere.00344-16 PMCID: PMC5177732  Chung The H, Boinett C, Pham Thanh D, Jenkins C, Weill FX, Howden BP, Valcanis M, De Lappe N, Cormican M, Wangchuk S, Bodhidatta L, Mason CJ, Nguyen TNT, Ha Thanh T, Voong VP, Duong VT, Nguyen PHL, Turner P, Wick R, Ceyssens PJ, Thwaites G, Holt KE, Thomson NR, Rabaa MA, Baker S. Dissecting the molecular evolution of fluoroquinolone-resistant *Shigella sonnei*. Nat Commun. 2019 Oct 23;10(1):4828. doi: 10.1038/s41467-019-12823-0. PubMed PMID: 31645551; PubMed Central PMCID: PMC6811581. |
| **Uzbekistan** | Madiyarov RS, Bektemirov AM, Ibadova GA, Abdukhalilova GK, Khodiev AV, Bodhidatta L, Sethabutr O, Mason CJ. Antimicrobial resistance patterns and prevalence of class 1 and 2 integrons in *Shigella* *flexneri* and *Shigella* *sonnei* isolated in Uzbekistan. Gut Pathog. 2010 Dec 9;2(1):18. doi: 10.1186/1757-4749-2-18. PubMed PMID: 21143880; PubMed Central PMCID: PMC3017001.  Chung The H, Boinett C, Pham Thanh D, Jenkins C, Weill FX, Howden BP, Valcanis M, De Lappe N, Cormican M, Wangchuk S, Bodhidatta L, Mason CJ, Nguyen TNT, Ha Thanh T, Voong VP, Duong VT, Nguyen PHL, Turner P, Wick R, Ceyssens PJ, Thwaites G, Holt KE, Thomson NR, Rabaa MA, Baker S. Dissecting the molecular evolution of fluoroquinolone-resistant *Shigella sonnei*. Nat Commun. 2019 Oct 23;10(1):4828. doi: 10.1038/s41467-019-12823-0. PubMed PMID: 31645551; PubMed Central PMCID: PMC6811581. |
| **Venezuela** | Sati HF, Bruinsma N, Galas M, Hsieh J, Sanhueza A, Ramon Pardo P, Espinal MA. Characterizing *Shigella* species distribution and antimicrobial susceptibility to ciprofloxacin and nalidixic acid in Latin America between 2000-2015. PLoS One. 2019 Aug 2;14(8):e0220445. doi: 10.1371/journal.pone.0220445. PubMed PMID: 31374081; PubMed Central PMCID: PMC6677304. |
| **Vietnam** | Chung The H, Boinett C, Pham Thanh D, Jenkins C, Weill FX, Howden BP, Valcanis M, De Lappe N, Cormican M, Wangchuk S, Bodhidatta L, Mason CJ, Nguyen TNT, Ha Thanh T, Voong VP, Duong VT, Nguyen PHL, Turner P, Wick R, Ceyssens PJ, Thwaites G, Holt KE, Thomson NR, Rabaa MA, Baker S. Dissecting the molecular evolution of fluoroquinolone-resistant *Shigella sonnei*. Nat Commun. 2019 Oct 23;10(1):4828. doi: 10.1038/s41467-019-12823-0. PubMed PMID: 31645551; PubMed Central PMCID: PMC6811581.  Chung The H, Rabaa MA, Pham Thanh D, De Lappe N, Cormican M, Valcanis M, Howden BP, Wangchuk S, Bodhidatta L, Mason CJ, Nguyen Thi Nguyen T, Vu Thuy D, Thompson CN, Phu Huong Lan N, Voong Vinh P, Ha Thanh T, Turner P, Sar P, Thwaites G, Thomson NR, Holt KE, Baker S. South Asia as a reservoir for the global spread of ciprofloxacin-resistant *Shigella sonnei*: A Cross-Sectional Study. PLoS Med. 2016 Aug 2;13(8):e1002055. doi: 10.1371/journal.pmed.1002055. PubMed PMID: 27483136; PubMed Central PMCID: PMC4970813. |

**Extended spectrum beta-lactamase producing**

| **Country** | **Reference** |
| --- | --- |
| **Argentina** | Radice M, Gonzéález C, Power P, Vidal MC, Gutkind G. Third-generation cephalosporin resistance in *Shigella sonnei*, Argentina. Emerg Infect Dis. 2001 May-Jun;7(3):442-3. PubMed PMID: 11384523; PubMed Central PMCID: PMC2631805. |
| **Australia** | Williams E, Lew TE, Fuller A, Spelman DW, Jenney AW. A case of multi-drug resistant ESBL-producing *Shigella sonnei* acute acalculous cholecystitis and gastroenteritis in a returned traveller. J Travel Med. 2018 Aug 1;25(1). doi: 10.1093/jtm/tay029. PubMed PMID: 30169854.  Ingle DJ, Easton M, Valcanis M, Seemann T, Kwong JC, Stephens N, Carter GP, Gonçalves da Silva A, Adamopoulos J, Baines SL, Holt KE, Chow EPF, Fairley CK, Chen MY, Kirk MD, Howden BP, Williamson DA. Co-circulation of multidrug-resistant *Shigella* among men who have sex with men in Australia. Clin Infect Dis. 2019 Oct 15;69(9):1535-1544. doi: 10.1093/cid/ciz005. PubMed PMID: 30615105. |
| **Bangladesh** | Rahman M, Shoma S, Rashid H, Siddique AK, Nair GB, Sack DA. Extended-spectrum beta-lactamase-mediated third-generation cephalosporin resistance in *Shigella* isolates in Bangladesh. J Antimicrob Chemother. 2004 Oct;54(4):846-7. PubMed PMID: 15329365. |
| **Belgium** | Vrints M, Mairiaux E, Van Meervenne E, Collard JM, Bertrand S. Surveillance of antibiotic susceptibility patterns among *Shigella sonnei* strains isolated in Belgium during the 18-year period 1990 to 2007. J Clin Microbiol. 2009 May;47(5):1379-85. doi: 10.1128/JCM.02460-08. Epub 2009 Mar 25. PubMed PMID: 19321731; PubMed Central PMCID: PMC2681836. |
| **Bhutan** | Chung The H, Rabaa MA, Thanh DP, Ruekit S, Wangchuk S, Dorji T, Tshering KP, Nguyen TNT, Vinh PV, Thanh TH, Minh CNN, Turner P, Sar P, Thwaites G, Holt KE, Thomson NR, Bodhidatta L, Jeffries Mason C, Baker S. Introduction and establishment of fluoroquinolone-resistant *Shigella sonnei* into Bhutan. Microb Genom. 2015 Dec 24;1(6):e000042. doi: 10.1099/mgen.0.000042. PubMed PMID: 28348825; PubMed Central PMCID: PMC5320628.  Chung The H, Boinett C, Pham Thanh D, Jenkins C, Weill FX, Howden BP, Valcanis M, De Lappe N, Cormican M, Wangchuk S, Bodhidatta L, Mason CJ, Nguyen TNT, Ha Thanh T, Voong VP, Duong VT, Nguyen PHL, Turner P, Wick R, Ceyssens PJ, Thwaites G, Holt KE, Thomson NR, Rabaa MA, Baker S. Dissecting the molecular evolution of fluoroquinolone-resistant *Shigella sonnei*. Nat Commun. 2019 Oct 23;10(1):4828. doi: 10.1038/s41467-019-12823-0. PubMed PMID: 31645551; PubMed Central PMCID: PMC6811581. |
| **Brazil** | Freitas DY, Araújo S, Folador ARC, Ramos RTJ, Azevedo JSN, Tacão M, Silva A, Henriques I, Baraúna RA. Extended spectrum beta-lactamase-producing Gram-negative bacteria recovered from an Amazonian lake near the city of Belém, Brazil. Front Microbiol. 2019 Feb 28;10:364. doi: 10.3389/fmicb.2019.00364. PubMed PMID: 30873145; PubMed Central PMCID: PMC6403167. |
| **Cambodia** | Poramathikul K, Bodhidatta L, Chiek S, Oransathid W, Ruekit S, Nobthai P, Lurchachaiwong W, Serichantalergs O, Lon C, Swierczewski B. Multidrug-resistant *Shigella* infections in patients with diarrhea, Cambodia, 2014-2015. Emerg Infect Dis. 2016 Sep;22(9):1640-3. doi: 10.3201/eid2209.152058. PubMed PMID: 27532684; PubMed Central PMCID: PMC4994341. |
| **China** | Ma Q, Xu X, Luo M, Wang J, Yang C, Hu X, Liang B, Wu F, Yang X, Wang J, Liu H, Li W, Zhong Y, Li P, Xie J, Jia L, Wang L, Hao R, Du X, Qiu S, Song H, Sun Y. A Waterborne Outbreak of *Shigella* *sonnei* with resistance to azithromycin and third-generation cephalosporins in China in 2015. Antimicrob Agents Chemother. 2017 May 24;61(6). pii: e00308-17. doi: 10.1128/AAC.00308-17. PubMed PMID: 28373192; PubMed Central PMCID: PMC5444132.  Zhang CL, Liu QZ, Wang J, Chu X, Shen LM, Guo YY. Epidemic and virulence characteristic of *Shigella* *spp*. with extended-spectrum cephalosporin resistance in Xiaoshan District, Hangzhou, China. BMC Infect Dis. 2014 May 15;14:260. doi: 10.1186/1471-2334-14-260. PubMed PMID: 24886028; PubMed Central PMCID: PMC4229937. |
| **Colombia** | Baker KS, Campos J, Pichel M, Della Gaspera A, Duarte-Martínez F, Campos-Chacón E, Bolaños-Acuña HM, Guzmán-Verri C, Mather AE, Diaz Velasco S, Zamudio Rojas ML, Forbester JL, Connor TR, Keddy KH, Smith AM, López de Delgado EA, Angiolillo G, Cuaical N, Fernández J, Aguayo C, Morales Aguilar M, Valenzuela C, Morales Medrano AJ, Sirok A, Weiler Gustafson N, Diaz Guevara PL, Montaño LA, Perez E, Thomson NR. Whole genome sequencing of *Shigella sonnei* through PulseNet Latin America and Caribbean: advancing global surveillance of foodborne illnesses. Clin Microbiol Infect. 2017 Nov;23(11):845-853. doi: 10.1016/j.cmi.2017.03.021. PubMed PMID: 28389276; PubMed Central PMCID: PMC5667938. |
| **France** | Lefort A, Arlet G, Join-Lambert OF, Lecuit M, Lortholary O. Novel extended-spectrum beta-lactamase in *Shigella sonnei*. Emerg Infect Dis. 2007 Apr;13(4):653-4. PubMed PMID: 17561568; PubMed Central PMCID: PMC2725971. |
| **Germany** | Hoffmann C, Sahly H, Jessen A, Ingiliz P, Stellbrink HJ, Neifer S, Schewe K, Dupke S, Baumgarten A, Kuschel A, Krznaric I. High rates of quinolone-resistant strains of *Shigella sonnei* in HIV-infected MSM. Infection. 2013 Oct;41(5):999-1003. doi: 10.1007/s15010-013-0501-4. PubMed PMID: 23852945. |
| **Hong Kong** | Cheung TK, Chu YW, Tsang GK, Ngan JY, Hui IS, Kam KM. Emergence of CTX-M-type beta-lactam resistance in *Shigella spp*. in Hong Kong. Int J Antimicrob Agents. 2005 Apr;25(4):350-2. PubMed PMID: 15784319. |
| **India** | Bhattacharya D, Bhattacharjee H, Ramanathan T, Sudharma SD, Singhania M, Sugunan AP, Roy S. Third-generation cephalosporin resistance in clinical isolate of *Shigella sonnei* in Andaman & Nicobar Islands, India. J Infect Dev Ctries. 2011 Sep 14;5(9):674-6. PubMed PMID: 21918310.  Madhavan A, Balakrishnan S, Vasudevapanicker J. Antibiotic susceptibility pattern of *Shigella* isolates in a tertiary healthcare center. J Lab Physicians. 2018 Apr-Jun;10(2):140-144. doi: 10.4103/JLP.JLP_93_17. PubMed PMID: 29692577; PubMed Central PMCID: PMC5896178.  Chung The H, Boinett C, Pham Thanh D, Jenkins C, Weill FX, Howden BP, Valcanis M, De Lappe N, Cormican M, Wangchuk S, Bodhidatta L, Mason CJ, Nguyen TNT, Ha Thanh T, Voong VP, Duong VT, Nguyen PHL, Turner P, Wick R, Ceyssens PJ, Thwaites G, Holt KE, Thomson NR, Rabaa MA, Baker S. Dissecting the molecular evolution of fluoroquinolone-resistant *Shigella sonnei*. Nat Commun. 2019 Oct 23;10(1):4828. doi: 10.1038/s41467-019-12823-0. PubMed PMID: 31645551; PubMed Central PMCID: PMC6811581. |
| **Iran** | Khademi F, Sahebkar A. Fluoroquinolones-resistant *Shigella* species in Iranian children: a meta-analysis. World J Pediatr. 2019 Oct;15(5):441-453. doi: 10.1007/s12519-019-00263-1. Epub 2019 Jun 1. PubMed PMID: 31154582.  Ranjbar R, Ghazi FM, Farshad S, Giammanco GM, Aleo A, Owlia P, Jonaidi N, Sadeghifard N, Mammina C. The occurrence of extended-spectrum β-lactamase producing *Shigella* *spp*. in Tehran, Iran. Iran J Microbiol. 2013 Jun;5(2):108-12. PubMed PMID: 23825726; PubMed Central PMCID: PMC3696844. |
| **Israel** | Vasilev V, Japheth R, Yishai R, Andorn N, Valinsky L, Navon-Venezia S, Chmelnitsky I, Carmeli Y, Cohen D. Extended-spectrum beta-lactamase-producing *Shigella strains* in Israel, 2000-2004. Eur J Clin Microbiol Infect Dis. 2007 Mar;26(3):189-94. PubMed PMID: 17265070. |
| **Lebanon** | Sabra AH, Araj GF, Kattar MM, Abi-Rached RY, Khairallah MT, Klena JD, Matar GM. Molecular characterization of ESBL-producing *Shigella sonnei* isolates from patients with bacilliary dysentery in Lebanon. J Infect Dev Ctries. 2009 May 1;3(4):300-5. PubMed PMID: 19759494. |
| **New Zealand** | Heffernan H, Woodhouse R, Hewison C, Sherwood J. Antimicrobial resistance among *Shigella* in New Zealand. N Z Med J. 2018 Jun 22;131(1477):56-62. PubMed PMID: 29927916. |
| **South Korea** | Kim JS, Kim JJ, Kim SJ, Jeon SE, Seo KY, Choi JK, Kim NO, Hong S, Chung GT, Yoo CK, Kim YT, Cheun HI, Bae GR, Yeo YH, Ha GJ, Choi MS, Kang SJ, Kim J. Outbreak of ciprofloxacin-resistant *Shigella sonnei* associated with travel to Vietnam, Republic of Korea. Emerg Infect Dis. 2015 Jul;21(7):1247-50. doi: 10.3201/eid2107.150363. PubMed PMID: 26079171; PubMed Central PMCID: PMC4480405.  Pai H, Choi EH, Lee HJ, Hong JY, Jacoby GA. Identification of CTX-M-14 extended-spectrum beta-lactamase in clinical isolates of *Shigella sonnei*, *Escherichia coli*, and *Klebsiella pneumoniae* in Korea. J Clin Microbiol. 2001 Oct;39(10):3747-9. PubMed PMID: 11574608; PubMed Central PMCID: PMC88424.  Kim S, Kim J, Kang Y, Park Y, Lee B. Occurrence of extended-spectrum beta-lactamases in members of the genus *Shigella* in the Republic of Korea. J Clin Microbiol. 2004 Nov;42(11):5264-9. PubMed PMID: 15528724; PubMed Central PMCID: PMC525143. |
| **Switzerland** | Nüesch-Inderbinen M, Heini N, Zurfluh K, Althaus D, Hächler H, Stephan R. *Shigella* antimicrobial drug resistance mechanisms, 2004-2014. Emerg Infect Dis. 2016 Jun;22(6):1083-5. doi: 10.3201/eid2206.152088. PubMed PMID: 27191035; PubMed Central PMCID: PMC4880098. |
| **Taiwan** | Huang IF, Chiu CH, Wang MH, Wu CY, Hsieh KS, Chiou CC. Outbreak of dysentery associated with ceftriaxone-resistant *Shigella sonnei*: First report of plasmid-mediated CMY-2-type AmpC beta-lactamase resistance in S. sonnei. J Clin Microbiol. 2005 Jun;43(6):2608-12. PubMed PMID: 15956372; PubMed Central PMCID: PMC1151904. |
| **Turkey** | Acikgoz ZC, Gulay Z, Bicmen M, Gocer S, Gamberzade S. CTX-M-3 extended-spectrum beta-lactamase in a *Shigella sonnei* clinical isolate: first report from Turkey. Scand J Infect Dis. 2003;35(8):503-5. PubMed PMID: 14514153.  Kacmaz B, Unaldi O, Sultan N, Durmaz R. Drug resistance profiles and clonality of sporadic *Shigella sonnei* isolates in Ankara, Turkey. Braz J Microbiol. 2014 Oct 9;45(3):845-9. eCollection 2014. PubMed PMID: 25477917; PubMed Central PMCID: PMC4204968. |
| **United Kingdom** | Mook P, McCormick J, Bains M, Cowley LA, Chattaway MA, Jenkins C, Mikhail A, Hughes G, Elson R, Day M, Manuel R, Dave J, Field N, Godbole G, Dallman T, Crook P. ESBL-producing and macrolide-resistant *Shigella sonnei* infections among men who have sex with men, England, 2015. Emerg Infect Dis. 2016 Nov;22(11):1948-1952. doi: 10.3201/eid2211.160653. PubMed PMID: 27767929; PubMed Central PMCID: PMC5088027. |
| **Vietnam** | Nguyen NT, Ha V, Tran NV, Stabler R, Pham DT, Le TM, van Doorn HR, Cerdeño-Tárraga A, Thomson N, Campbell J, Nguyen VM, Tran TT, Pham MV, Cao TT, Wren B, Farrar J, Baker S. The sudden dominance of blaCTX-M harbouring plasmids in *Shigella spp*. Circulating in Southern Vietnam. PLoS Negl Trop Dis. 2010 Jun 8;4(6):e702. doi: 10.1371/journal.pntd.0000702. PubMed PMID: 20544028; PubMed Central PMCID: PMC2882334.  Darton TC, Tuyen HT, The HC, Newton PN, Dance DAB, Phetsouvanh R, Davong V, Campbell JI, Hoang NVM, Thwaites GE, Parry CM, Thanh DP, Baker S. Azithromycin resistance in *Shigella* *spp*. in southeast Asia. Antimicrob Agents Chemother. 2018 Mar 27;62(4). pii: e01748-17. doi: 10.1128/AAC.01748-17. PubMed PMID: 29378707; PubMed Central PMCID: PMC5913960.  Chung The H, Boinett C, Pham Thanh D, Jenkins C, Weill FX, Howden BP, Valcanis M, De Lappe N, Cormican M, Wangchuk S, Bodhidatta L, Mason CJ, Nguyen TNT, Ha Thanh T, Voong VP, Duong VT, Nguyen PHL, Turner P, Wick R, Ceyssens PJ, Thwaites G, Holt KE, Thomson NR, Rabaa MA, Baker S. Dissecting the molecular evolution of fluoroquinolone-resistant *Shigella sonnei*. Nat Commun. 2019 Oct 23;10(1):4828. doi: 10.1038/s41467-019-12823-0. PubMed PMID: 31645551; PubMed Central PMCID: PMC6811581. |

**Reduced sensitivity to azithromycin**

| **Country** | **Reference** |
| --- | --- |
| **Australia** | Ingle DJ, Easton M, Valcanis M, Seemann T, Kwong JC, Stephens N, Carter GP, Gonçalves da Silva A, Adamopoulos J, Baines SL, Holt KE, Chow EPF, Fairley CK, Chen MY, Kirk MD, Howden BP, Williamson DA. Co-circulation of multidrug-resistant *Shigella* among men who have sex with men in Australia. Clin Infect Dis. 2019 Oct 15;69(9):1535-1544. doi: 10.1093/cid/ciz005. PubMed PMID: 30615105. |
| **Bangladesh** | Rahman M, Shoma S, Rashid H, Siddique AK, Nair GB, Sack DA. Extended-spectrum beta-lactamase-mediated third-generation cephalosporin resistance in *Shigella* isolates in Bangladesh. J Antimicrob Chemother. 2004 Oct;54(4):846-7. PubMed PMID: 15329365 |
| **Canada** | Yousfi K, Gaudreau C, Pilon PA, Lefebvre B, Walker M, Fournier É, Doualla Bell F, Martineau C, Longtin J, Bekal S. Genetic mechanisms behind the spread of reduced susceptibility to azithromycin in *Shigella* strains isolated from men who have sex with men in Québec, Canada. Antimicrob Agents Chemother. 2019 Jan 29;63(2). pii: e01679-18. doi: 10.1128/AAC.01679-18. PubMed PMID: 30455248; PubMed Central PMCID: PMC6355565. |
| **China** | Ma Q, Xu X, Luo M, Wang J, Yang C, Hu X, Liang B, Wu F, Yang X, Wang J, Liu H, Li W, Zhong Y, Li P, Xie J, Jia L, Wang L, Hao R, Du X, Qiu S, Song H, Sun Y. A Waterborne Outbreak of *Shigella* *sonnei* with resistance to azithromycin and third-generation cephalosporins in China in 2015. Antimicrob Agents Chemother. 2017 May 24;61(6). pii: e00308-17. doi: 10.1128/AAC.00308-17. PubMed PMID: 28373192; PubMed Central PMCID: PMC5444132. |
| **France** | Boumghar-Bourtchai L, Mariani-Kurkdjian P, Bingen E, Filliol I, Dhalluin A, Ifrane SA, Weill FX, Leclercq R. Macrolide-resistant *Shigella sonnei*. Emerg Infect Dis. 2008 Aug;14(8):1297-9. doi: 10.3201/eid1408.080147. PubMed PMID: 18680661; PubMed Central PMCID: PMC2600399. |
| **Iran** | Khademi F, Sahebkar A. Fluoroquinolones-resistant *Shigella* species in Iranian children: a meta-analysis. World J Pediatr. 2019 Oct;15(5):441-453. doi: 10.1007/s12519-019-00263-1. PubMed PMID: 31154582. |
| **Israel** | Ezernitchi AV, Sirotkin E, Danino D, Agmon V, Valinsky L, Rokney A. Azithromycin non-susceptible *Shigella* circulating in Israel, 2014-2016. PLoS One. 2019 Oct 18;14(10):e0221458. doi: 10.1371/journal.pone.0221458. PubMed PMID: 31626667; PubMed Central PMCID: PMC6799884. |
| **New Zealand** | Heffernan H, Woodhouse R, Hewison C, Sherwood J. Antimicrobial resistance among *Shigella* in New Zealand. N Z Med J. 2018 Jun 22;131(1477):56-62. PubMed PMID: 29927916. |
| **Switzerland** | Nüesch-Inderbinen M, Heini N, Zurfluh K, Althaus D, Hächler H, Stephan R. *Shigella* antimicrobial drug resistance mechanisms, 2004-2014. Emerg Infect Dis. 2016 Jun;22(6):1083-5. doi: 10.3201/eid2206.152088. PubMed PMID: 27191035; PubMed Central PMCID: PMC4880098. |
| **United Kingdom** | Mook P, McCormick J, Bains M, Cowley LA, Chattaway MA, Jenkins C, Mikhail A, Hughes G, Elson R, Day M, Manuel R, Dave J, Field N, Godbole G, Dallman T, Crook P. ESBL-producing and macrolide-resistant *Shigella sonnei* infections among men who have sex with men, England, 2015. Emerg Infect Dis. 2016 Nov;22(11):1948-1952. doi: 10.3201/eid2211.160653. PubMed PMID: 27767929; PubMed Central PMCID: PMC5088027. |
| **United States** | Howie RL, Folster JP, Bowen A, Barzilay EJ, Whichard JM. Reduced azithromycin susceptibility in *Shigella sonnei*, United States. Microb Drug Resist. 2010 Dec;16(4):245-8. doi: 10.1089/mdr.2010.0028. PubMed PMID: 20624094.  Sjölund Karlsson M, Bowen A, Reporter R, Folster JP, Grass JE, Howie RL, Taylor J, Whichard JM. Outbreak of infections caused by *Shigella sonnei* with reduced susceptibility to azithromycin in the United States. Antimicrob Agents Chemother. 2013 Mar;57(3):1559-60. doi: 10.1128/AAC.02360-12. PubMed PMID: 23274665; PubMed Central PMCID: PMC3591876. |
| **Vietnam** | Darton TC, Tuyen HT, The HC, Newton PN, Dance DAB, Phetsouvanh R, Davong V, Campbell JI, Hoang NVM, Thwaites GE, Parry CM, Thanh DP, Baker S. Azithromycin resistance in *Shigella* *spp*. in southeast Asia. Antimicrob Agents Chemother. 2018 Mar 27;62(4). pii: e01748-17. doi: 10.1128/AAC.01748-17. PubMed PMID: 29378707; PubMed Central PMCID: PMC5913960. |
